# Tic Douloureux of the Face and Head

**Published:** 1844-06

**Authors:** 


### PRACTICAL MEDICINE, &c.

*Tic Douloureux of the Face and Head.*—M. Ducros recently communicated to the Academy the details of some well marked cases of this distressing disease, which were rapidly cured by the use of strong ammonia, applied to the palate, gums, &c., with a camel-hair brush, so as to occasion a profuse discharge of tears and saliva. He requested the physicians of several of the metropolitan hospitals to repeat his experiments on a large scale; and the results of their trials have been, he says, most satisfactory.

(The strong Aqua or Liquor Ammoniæ, taken internally, will be found to be a most valuable remedy in many cases of neuralgic suffering about the face and head, odontalgia, severe nervous headache, &c. The best mode of administering it is to mix from 20 to 40 drops in a cupful of very thick gruel, and to take this at bed-time, or whenever the paroxysm of pain is present. The ammonia must be well blended with the gruel, else it will irritate very painfully the inside of the mouth and throat. It should produce profuse salivation and lachrymation. In very severe and obstinate cases, it may be applied outwardly at the same time.)—*Med. Examiner, from Medico-Chirurgical Review.*

We subjoin some remarks of Dr. Watson, upon the same subject.

Mr. Abernethy used to relate, in his lectures, many instances of tic which he had been successful in curing by measures which were solely directed to the improvement of the stomach and bowels. He had a notion, that in patients who suffer under this disorder, there were always two functions wrong; those of the nervous system on the one hand, those of the digestive system on the other. And I am sure you will commonly find indications of a faulty state of both these systems. "The two," he used to say, "were the common parents of a numerous progeny of very dissimilar local diseases. In tic douloureux, you must seek to put the digestive organs right, or to soothe the nervous system, according as the one or the other may seem to be the principal and primary cause of the disease. Take away one of the parents, and there will be no more propagation."

In these cases, the unhealthy state of the digestive apparatus may be marked by obvious signs; a furred tongue, loss of appetite, costive bowels; or it may reveal itself by no other symptoms than the pain. It may depend upon the mere presence of acid in the stomach. Dr. Rigby tells us that having suffered in his own person an intense attack of tic douloureux, which opium did not assuage, he swallowed, at the suggestion of a friend, some carbonate of soda dissolved in water. The effect was almost immediate: carbonic acid was erucated, and the pain quickly abated. More often the cause of offence appears to lie in some part of the intestines; and purgatives do good. Sir C. Bell—drawing a bow at a venture, achieved the cure of a patient, upon whom much previous treatment had been expended in vain, by some pills composed of cathartic extract, croton oil, and galbanum. He mixes one, or two, drops of the oleum tiglii, with a drachm of the compound extract of colocynth; and gives five grains of this mass, with ten grains of the compound galbanum pill, at bed-time. I mention the exact proportions and dose, because other cases have been since reported, both by Sir Charles and others, in which the same prescription was followed with the same success.

When the disease occurs in a rheumatic individual, and especially when, as is sometimes the case, it alternates with rheumatism of other tissues, the remedies which have been found useful in rheumatism deserve a fair trial: guiacum; colchicum; calomel and opium.

When all has been done that can be done towards restoring or improving the general health, we may turn our thoughts to local remedies. It is plain that these must be inefficient when the local pain results from constitutional causes that are unredressed or perhaps incurable. Yet even then topical measures may soothe the pain for a while. * * *

There is a kind of face-*ache*, which cannot properly be included as a species of neuralgia, for it does not occur in short stabbing paroxysms, nor is the pain acute enough to entitle it to the name of tic douloureux; but which is very common, very distressing, and under ordinary treatment sometimes very intractable. It is called often a rheumatic pain; it occupies the lower part of the face, the jaw principally, and the patient cannot say exactly whereabouts it is most intense. It is often thought to proceed from toothache, and bad or suspected teeth are extracted, but with no good effect. Now I allude to this for the sake of saying that some years ago I was told by an experienced old apothecary, that this face-ache might be almost always and speedily cured by the muriate of ammonia;—a medicine that we seldom give internally here, although it is so much used in Germany. And I have again and again availed myself of this hint, and been much thanked by my patients for the good I did them with this muriate of ammonia. It does not *always* succeed; but it *often* does. It should be given in half drachm doses, dissolved in water, or in almost any vehicle, three or four times a day. If the pain does not yield after four doses, you may cease to expect any benefit from it. In two or three instances of a similar kind that I have recently had to treat, I have found the iodide of potassium, in doses of five or six grains, work a speedy and permanent cure. This induces me to suppose that the pain in some of these cases is periosteal; judging from the ascertained efficacy of the iodide in other periosteal affections attended with pain.—*Watson's Lectures.*

